# Explicit Sequence Memory in Recall of Temporally-structured Episodes

**DOI:** 10.1038/s41598-020-59472-8

**Published:** 2020-02-14

**Authors:** Yonatan Stern, Ron Katz, Talya Sadeh

**Affiliations:** 1 The Department of Cognitive and Brain Sciences, Ben-Gurion University of the Negev, Beer Sheva, Israel; 2 Zlotowski Center for Neuroscience, Ben-Gurion University of the Negev, Beer Sheva, Israel; 3 Department of Psychology, Ben-Gurion University of the Negev, Beer Sheva, Israel; 4 Department of Psychology, University of Haifa, Haifa, Israel

**Keywords:** Hippocampus, Long-term memory

## Abstract

The order in which events unfold over time is an important scaffold aiding recollection. This study asks whether explicit order memory is enhanced for items sharing similar internally-driven temporal contexts. To tap internally-driven temporal context, we capitalized on the Temporal Contiguity Effect whereby recollection of one item promotes recall of adjacently-encoded items. We compared pairs encoded and retrieved contiguously (cont-enc-ret), whose items share internally-driven temporal contexts, to pairs retrieved, but not encoded, contiguously (cont-ret) and to pairs encoded, but not retrieved, contiguously (cont-enc). Cont-enc-ret pairs exhibited superior relative order over cont-ret pairs, supporting accounts emphasizing shared temporal context as opposed to temporal distinctiveness in driving sequence memory. No difference was found in absolute order between the pair types, in line with theories suggesting a dissociation between relative and absolute order. Additionally, cont-enc-ret and cont-enc pairs exhibited equivalent relative order, supporting the role of encoding as opposed to retrieval in the enhancement of relative order. Finally, cont-enc-ret pairs were perceived as closer than cont-enc pairs, supporting the claim that cont-enc-ret pairs constitute part of a temporally-coherent episode. Together, these results implicate internally-driven temporal context in the formation of temporally-structured episodes that enhances sequence memory of the items within the episode.

## Introduction

When we remember an episode from our past, we do not merely recollect its details in random order, rather the sequence in which the episode unfolded over time provides an important scaffold for recollection. Recalling one item brings to mind other items that occurred adjacently in time^1,2^. In the absence of such a temporal structure, an episode would amount to no more than a random list of details. ‘Mental time travel’, the ability to re-experience past episodes^3,4^ and the ability to simulate future episodes using past events as templates^5^ both rely on the coherence provided by recollection of sequence memory^6^. Furthermore, the order of events often defines the meaning and significance that we attribute to them^7^. For example, the ability to infer a causal relation between two events, requires remembering that the cause preceded the effect^2^. Thus, explicit sequence memory plays an important role in constructing a coherent representation of one’s past experiences.

Past studies have found enhanced sequence memory^8^ and an increase in the perceived proximity^9^ between items sharing the same experimentally-manipulated context. Inspired by these findings, the focus of the current study is on the role of internally-driven temporal context and its effects on different types of sequence memory. Internally-driven temporal context is best manifested by the seminal Temporal Contiguity Effect (TCE) in free recall paradigms. This effect pertains to the tendency to contiguously recall items that were temporally proximal during the study phase^10,11^. The TCE is accounted for by the similar internally-driven temporal context of items studied contiguously. Reinstatement of this context at test is thus more likely to trigger recall of items studied contiguously than of items studied further apart^12^. Thus, items that were studied contiguously might share similar internally-driven contexts, but contiguity at both encoding and retrieval is an even stronger indication of reliance on internally-driven context.

Utilizing the TCE as a measure of internally-driven context, we examined whether contiguous encoding and recall enhances explicit memory for the sequence of items. Furthermore, as we explain below, we sought to tease apart the contribution of contiguous encoding as opposed to contiguous recall on sequence memory.

Two major competing theoretical approaches have been offered to account for the effects of temporal distance and context on sequence memory^13^. The first, henceforth referred to as the Distinctiveness Approach, highlights the role of increased temporal distance and distinctiveness in promoting sequence memory^14,15^. Within this theoretical approach, different accounts of temporal distinctiveness have been proposed, such as gradual changes in the temporal representation of items during encoding^16,17^, or the binding of items to their list position^14,15^. Importantly, these accounts converge on the prediction that more distal items have more distinct representations, whereas proximal items have more overlapping representations. This increased distinctiveness of distal items, in turn, enhances memory for their recency and relative order, a finding that has been widely replicated for review see^13,18,19^.

On the other hand, the second theoretical approach, henceforth referred to as the Shared Context Approach, posits that temporal proximity and shared context between adjacent items support sequence memory. Within this approach, associative chaining theories, typically applied to the recall of serial order, emphasize the role of inter-item associations in sequence memory, with the recall of one item cuing adjacent items^20,21^. Likewise, hierarchal models, such as Farrell’s (2012) ^22^, posit that adjacent items are bound together into clusters and within these clusters recall proceeds in a sequential manner. In a similar vein, theories of event cognition^2^ view temporal proximity between items as a prerequisite for their segmentation into an event, that subsequently enhances sequence memory for items within the event^23^. Finally, although not specific to sequence memory, the Temporal Context Model conceives of the overlap in contextual representations between adjacent items as a primary driving force in memory retrieval^12^. Thus, the common theme of these different accounts is that temporal proximity and shared temporal context is the basis for the binding of items that subsequently enhances their sequence memory. This is in contrast to the accounts within the Distinctiveness Approach above that emphasize the role of temporal distance and distinctiveness in supporting sequence memory. It should be noted that the some of the different theories discussed in brief here originate in different memory paradigms. Accordingly, the divergent effects of temporal distance are not necessarily contradictory.

Indeed, a study by DuBrow & Davachi (2013) demonstrated that each of these two approaches has its unique contribution and both are required to fully account for a variety of findings concerning sequence memory. In their study they examined the effects of experimentally-manipulated external context on sequence memory, by alternating stimuli category and encoding task during list presentation. On the one hand, they found that when presentation rates were long and relational encoding was encouraged, adjacent items sharing the same context exhibited superior relative order compared to adjacent items with different contexts (see Experiment 1). This finding is in line with the Shared Context Approach. In contrast, when item-related encoding (rather than relational encoding) was encouraged by introducing a between-item distractor task and quick presentation rates, temporal distance between items significantly improved relative order memory, while the effects of shared context between adjacent items disappeared (see Experiment 2). These latter results support the Distinctiveness Approach. Taken together, the results demonstrate that both approaches are required to account for sequence memory performance. The Shared Context Approach accounts for sequence memory performance when encoding relies on relational process—namely, when the encoding task highlights the creation of associations between items and is relatively lengthy. In contrast, the Distinctiveness Approach accounts for sequence memory performance when encoding relies on item-related processing, such as when the time allotted for encoding does not enable creation of associations between items, but only the processing of low-level features of the item itself.

Inspired by these findings, in the current study we examined the role of internally-driven temporal context (as opposed to externally-manipulated context) in shaping sequence memory. As mentioned, we capitalized on the TCE in free recall to operationalize temporal context. Given that performance in free recall relies heavily on relational processing both at encoding and at retrieval, we expected our results to align with the Shared Context Approach (rather than with the Distinctiveness Approach). Namely, we hypothesized that sequence memory would be enhanced for items sharing similar temporal contexts than for those with distinct temporal contexts. This hypothesis is in contrast to a wealth of prior literature showing the opposite effect, namely that temporal distance enhances sequence memory^18,19^.

Furthermore, in the current study we expanded previous work by examining the contribution of encoding and retrieval processes to sequence memory. Two lines of evidence point to the larger contribution of encoding, compared to retrieval, to sequence memory. First, the above-mentioned findings demonstrated that sequence memory was affected by manipulations of encoding processes. Specifically, both the encoding context (shared vs. distinct) and the encoding task (emphasizing relational- vs. item-related processing) affected performance in the sequence memory task^8^. Second, a large body of work^24,25^ has demonstrated that, as compared to retrieval, manipulating the deployment of attention during encoding has much larger effects on memory performance. While this line of work did not examine sequence memory, it provides strong evidence for the larger contribution of encoding than retrieval processes to memory performance.

We have thus far focused mostly on the effects of context on memory for relative order. However, the term ‘sequence memory’, encompasses an additional, distinct type of sequence: absolute order^13^. A further goal of the current study was to examine both types of sequence memory to paint a more detailed picture of the effects of context on sequence memory. While relative order refers to the relation *between* two items, i.e. recalling which item appeared first, absolute order refers to memory of the temporal position of an event *within* a broader timescale (i.e. its serial position). We examined both types of sequence memory, in search of a dissociation between these two types of order. We expected pairs of items encoded and recalled contiguously, as manifested by the TCE, to exhibit superior relative order over items that were not encoded contiguously but were recalled contiguously. In contrast, we did not expect a difference between the two categories in absolute order.

Three lines of research support this dissociation. First, accumulating evidence from neuroimaging^17,26^ and animal studies^16,27^ demonstrates that the two types of order information (relative and absolute) rely on distinct brain regions. Relative order is supported by the hippocampus and absolute order is supported by frontal brain regions. In addition, recent imaging studies have strongly implicated the hippocampus in the reinstatement of the encoding context thus supporting the TCE^28,29^. Together, these findings support the association between contiguous encoding and relative order in particular. Second, computational accounts of hierarchal memory such as Farrell’s (2012^22^) predict a dissociation between the two types of order memory. In his account, items within a cluster are bound together into a memory representation that has its own unique context. Within each cluster, recall proceeds in a serial manner relying on the items’ temporal sequence^22^. In contrast, transitions between clusters do not necessarily proceed in a sequential manner; rather they rely on various relations between the clusters’ representations such as source memory or semantic knowledge^12,22^. Based on this account, relative order is posited to play a central role in recall but only when examining items within the same cluster. In contrast, absolute order should generally be equal for items within and across clusters because of the non-serial transitions between clusters. Finally, the two approaches discussed above, the Shared Context Approach and the Distinctiveness Approach may account for performance in relative and absolute order memory respectively. The Shared Context Approach appears to account for sequence memory performance when encoding relies on relational process and thus concerns the relation between two items—namely, relative order. Accordingly, contiguously encoded items (sharing similar temporal contexts) are expected to show an advantage in relative order as compared to items not encoded contiguously. In contrast, the Distinctiveness Approach appears to account for sequence memory performance when encoding relies on item-related processes and measures pertaining to the item in isolation (rather than to its relation with other items). Thus, sequence memory in this case refers to absolute, rather than relative, order. Because item-related processing should not differ between contiguously and non-contiguously encoded items, we expected to find no difference in absolute order between them.

In the current study we used a novel paradigm that combines free recall with an ordering task. Pairs of items encoded and retrieved contiguously–cont-enc-ret—were compared with pairs of items retrieved but not encoded contiguously—cont-ret—allowing us to discern the contribution of contiguous encoding. We further characterized the contribution of encoding as opposed to recall processes to sequence memory, by comparing pairs encoded contiguously and retrieved non-contiguously–cont-enc–to cont-enc-ret pairs.

Seven predictions concerning the effects of internally-driven temporal context on explicit sequence memory were tested.

Our first three predictions were based on previous findings^8^, supporting the Shared Context Approach, which showed enhanced relative order for items sharing similar experimentally-manipulated encoding contexts versus those which do not share similar contexts.

## Prediction 1

We predicted that cont-enc-ret pairs would show superior relative order in comparison to cont-ret pairs.

## Prediction 2

We also examined relative order within the cont-ret category, as a function of the pair’s distance (i.e. lag) during encoding. If the Shared Context Approach provides a better account for relative order than the Distinctiveness Approach, we would expect that as distance decreases, relative order improves.

## Prediction 3

To further compare the two approaches, we examined the relation between distance scores and relative scores. The distance score is the difference between the distance between pair members during ordering and their actual distance during the study phase. A value of zero reflects correct assessment of distance, positive values reflect the ordering of pairs farther apart than their actual distance and negative values reflect the ordering of pairs closer to one another than their actual distance. If the Shared Context Approach provides a better account for relative order than the Distinctiveness Approach, we would expect that pairs that were perceived as close (i.e. small distance score), that is indicative of shared context, would exhibit increased relative order.

Our fourth and fifth predictions pertain to the expected dissociation between relative and absolute order. This dissociation was predicted based on the three lines of research described above.

## Prediction 4

We predicted that cont-enc-ret pairs would *not* show superior explicit absolute memory in comparison to cont-ret pairs.

## Prediction 5

Our fifth prediction was that between-subject correlations between relative order and the TCE magnitude would be greater than the correlation between absolute order and the TCE magnitude.

## Prediction 6

Our sixth prediction concerns the increased contribution of encoding as opposed to retrieval processes to relative order. To untangle the contributions of the retrieval and encoding processes, we compared cont-enc-ret pairs to items learned contiguously but recalled non-contiguously–cont-enc pairs. If superior relative order is a result of encoding, we would expect performance for cont-enc-ret pairs and cont-enc pairs to be equivalent, because both pair types were contiguously encoded. Alternatively, if relative order is a function of retrieval processes, we would expect superior relative order for the cont-enc-ret pairs over the cont-enc pairs.

## Prediction 7

Our final prediction concerns the ‘distance score’. We predicted that cont-enc-ret pairs would show enhanced distance memory (i.e. lower distance scores), in comparison to cont-enc pairs. Thus, contiguity at both encoding and retrieval more likely entails reliance on internally-driven context than contiguity at encoding alone. Such a result would further implicate internally-driven context in the formation of temporally-structured episodes^9^. This prediction is in line with findings that both the TCE^28,29^ and the binding of pair members into episodes are supported by the hippocampal system^30–32^.

**Figure 1:**
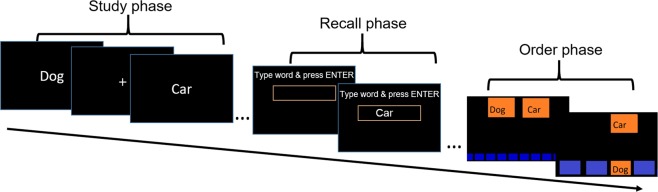
Experimental procedure for a single list in the two experiments. Participants were presented with 16 words successively (study phase), after which they had one minute to type as many words they could recall in any order (recall phase). In the no-order condition this constituted a single list, whereas in the order condition, recall was followed by an ordering task. Participants were presented with a pair of items recalled in the previous phase on the top of the screen. They were instructed to order each of the items appearing at the top of the screen, by dragging them, using the mouse, to the bottom of screen according to their initial position during the study phase. Participants had 13 s to perform the task, after which the screen was refreshed and a new pair was presented. The number of pairs presented in the order phase depended on the participants’ recall in the previous phase.

We tested these predictions in a series of two experiments, each including two conditions. In one condition there was only a standard free recall paradigm with a study and recall phase, and in the other condition the recall phase was followed by an ordering task. During each trial of the ordering task a pair of words that were previously recalled were presented, and participants ordered them according to their position during the study (see Fig. 1). Importantly, the pairs presented belonged to one of the categories discussed above: cont-enc-ret, cont-ret (Experiments 1 and 2), and cont-enc (Experiment 2).

## Results

### Prediction 1: Superior relative order for pairs encoded and retrieved contiguously (cont-enc-ret) over pairs not encoded contiguously (cont-ret)

In both experiments we found strong evidence for enhanced relative order for cont-enc-ret pairs in comparison to cont-ret pairs. In the first experiment the mean relative score of cont-enc-ret was 0.81 (SEM = 0.01), and the cont-ret mean was 0.64 (SEM = 0.17; see Table 1 for summary statistics of all order measures). A one-way repeated measures ANOVA (RMANOVA), including as covariates the differences between the two conditions in overall recall performance and in temporal score (see control analysis 1B below), revealed a significant effect of pair category on relative score (*F*_1,29_ = 18.89; *p* < 0.001; η²_p_ = 0.42). Likewise, a Bayesian RMANOVA revealed extremely strong evidence in favor of the main effect of pair category with a BF_10_ of > 21,000 in comparison to the null model that included the covariates as nuisance variables.

**Table 1:** Summary statistics of the ordering task from Experiments 1 & 2.

	Experiment 1		Experiment 2		
	cont-enc-ret	cont-ret	cont-enc-ret	cont-enc	cont-ret
Relative Score	0.81 (0.01)	0.64 (0.17)	0.80 (0.02)	0.77 (0.03)	0.65 (0.03)
Absolute Score	0.88 (0.01)	0.88 (0.01)	0.87 (0.01)	0.87 (0.02)	0.86 (0.02)
Absolute Deviation	1.27 (0.1)	1.3 (0.09)	1.33 (0.14)	1.33 (0.14)	1.5 (0.18)
Distance Score	0.41 (0.07)	−1.94 (0.21)	0.4 (0.1)	0.78 (0.9)	−1.25 (0.17)

Cont-enc-ret are items that were encoded and retrieved contiguously, whereas cont-ret are items that were not encoded contiguously but were retrieved contiguously. Cont-enc are items that were encoded contiguously but not retrieved contiguously. Scores are presented as Mean (SEM). Relative score is a measure of each pair’s relative ordering, regardless of the items’ absolute order. A score of one signifies completely accurate ordering. Absolute score and absolute deviation are measures of the first item’s ordering with regard to its unsigned deviation from its position during study. A score of one for absolute score signifies correct placement, and zero for absolute deviation signifies accurate ordering. Distance score is a measure of the distance between the two items, a score of zero indicates that they were placed in their original distance from each other, positive values indicate that they were placed farther apart than they were studied and negative values indicate that they were placed closer to each other than they were studied.

In Experiment 2 we replicated this finding. The mean relative score of cont-enc-ret was 0.80 (SEM = 0.02), the cont-ret mean was 0.65 (SEM = 0.03) and the cont-enc mean was 0.77 (SEM = 0.026). A one-way RMANOVA was performed to determine whether this difference is statistically significant. The assumption of sphericity was violated (*W* = 0.75; *p* < 0.05), so we used the Greenhouse-Geisser correction in the results reported below. The main effect of pair category was significant (*F*_1.6, 35.1_ = 9.4, *p* < 0.001, η²_p_ = 0.3). Post hoc comparisons revealed that cont-ret’s relative score was significantly lower than both cont-enc-ret (*t*_(24)_ = 4.44, *p*_*bonf*_ < 0.001) and cont-enc (*t*_(24)_ = 2.75, *p*_*bonf*_ < *0*.05). The equivalent Bayesian analyses yielded similar results. The evidence for the model including the main effect of pair type was extremely strong (BF_10_ = 209). Post hoc comparisons of cont-enc-ret and cont-ret were supported by strong evidence (BF_10_ = 164.5), and the comparison of cont-enc and cont-ret was supported by moderate evidence (BF_10_ = 4.38). To summarize, these two experiments yielded strong evidence that pairs encoded contiguously (cont-enc-ret and cont-enc) exhibit superior explicit memory for relative order over pairs encoded non-contiguously (cont-ret). Importantly, this finding is in contrast to the equivalent relative order found between items encoded contiguously (i.e., cont-enc-ret and cont-enc; see Prediction 6 below). In the following section we briefly discuss two control analyses that refute potentially confounding factors, their full discussion appears in Supplemental Material.

### Control analysis 1A: Confound of testing effect on relative score difference

**Figure 2:**
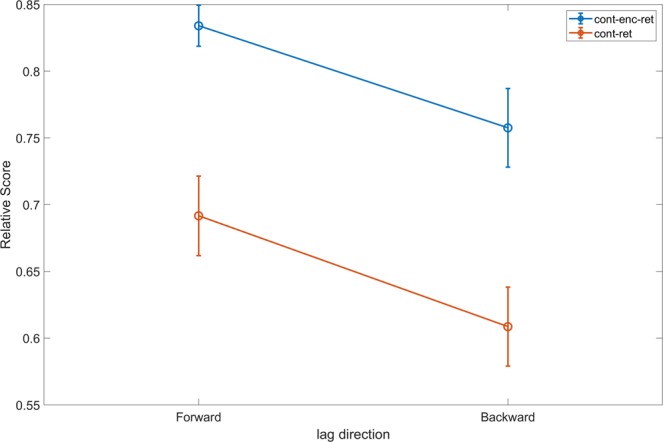
Relative score as a function of pair type and the transition direction. Both main effects of pair type (cont-enc-ret/ cont-ret) and direction (forward/ backward) are significant (*p* < 0.001, BF_10_ = 5*10^6^; p = 0.002, BF_10_ = 9.9 respectively), whereas the interaction is not (*p* > 0.8, BF_01_ = 4.05). Simple effects of pair type in both forward and backward direction are also significant (p < 0.001). Error bars denote SEM.

An alternative explanation for the increase in the cont-enc-ret relative score is that participants were not relying on the items’ position during the study phase but rather were ordering them according to their relative order during recall. This can be construed as a testing effect, whereby the recollection of items creates an enhanced memory trace of the testing phase^33^. Forward transitions (i.e. a positive lags) are more abundant in the cont-enc-ret than in the cont-ret category—a finding known as ‘forward asymmetry’ in the TCE^11^. That is, lag +1 are more likely than lag -1 recalls, whereas for greater lags positive and negative transitions are generally equally probable. In order to examine the possibility of a testing effect, we conducted a two-way RMANOVA with lag direction (forward/backward) and pair category (cont-enc-ret/cont-ret) as independent variables, relative score as the dependent variable, and experiment number as a covariate. To increase statistical power we combined the data from both experiments, and included in the analysis only participants who had at least 3 pairs in each of the four cells in order to ensure that values were based on sufficient observations (*N* = 41). We found a significant main effect of pair category (*F*_1,39_ = 29.9, *p* < 0.001, η²_p_ = 0.43), signifying a greater relative score for cont-enc-ret (mean = 0.80, SEM = 0.02) over cont-ret (mean = 0.65, SEM = 0.03). The main effect of direction was also significant (*F*_1,39_ = 11.1, *p* = 0.002, η^2^_p_ = 0.22), with forward transitions having a higher relative score (mean = 0.76, SEM = 0.03) than backward transitions (mean = 0.68, SEM = 0.03), in possible accordance with the testing effect interpretation. Importantly, however the interaction between pair type and direction was not significant (*F*_1,39_ <1, *p* > 0.8), indicating that the testing effect and the advantage of forward transitions did not differ between cont-enc-ret and cont-ret pairs (see Fig. 2). This was further corroborated by examining the simple effects of pair category as a function of direction. In both cases, cont-enc-ret pairs’ relative order was higher than cont-ret pairs’ (Simple effect of Forward, *F*_1,39_ = 18.5, *p* < 0.001; Backward, *F*_1,39_ = 14.8, *p* < 0.001). Accordingly, cont-enc-ret pairs’ increased accuracy cannot be solely explained by the testing effect, as is evident from the significant increase even in backward transitions where the testing effect is absent. These findings were further corroborated by Bayesian analyses (see Supplemental Material Section A).

### Control analysis 1B: Confound of condition type on relative score difference

An additional potential confound is that due to the inclusion of an ordering task following recall, recall was not performed ‘freely’ and was affected by the succeeding ordering task. In brief (see Supplemental Material Section C, Section F, and Supplemental Figs. 1 and 2), we reject this claim based on two findings. First, the results were replicated across the two experiments that employed both blocked and intermixed designs of the order of list type, where in the latter subjects could not predict the occurrence of an ordering task. Second, the findings were significant even after including as covariates the difference in overall recall performance and temporal scores between the two list conditions. Thus, changes in recall performance and utilization of temporal context following the introduction of the ordering task do not explain the variance that is captured by pair category.

### Prediction 2: Relative order as a function of temporal distance in cont-ret pairs

We examined relative score within cont-ret pairs as a function of the distance during encoding (i.e. lag). According to the Temporal Distinctiveness Approach, we would expect that pairs with greater lags would show enhanced relative order in comparison to pairs with shorter lags that presumably are less distinct from each other, whereas the Shared Context Approach predicts the opposite. For each subject we split the cont-ret pairs into terciles according to their lag. We included only subjects who had at least three pairs from each category (short/medium/long lag). To increase statistical power, we combined data from both experiments, and included experiment type as a covariate within the RMANOVA that was performed with lag as the independent variable, and relative score as the dependent variable.

**Figure 3:**
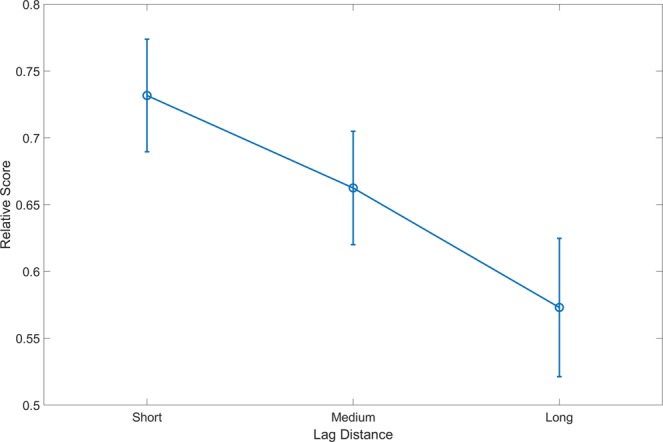
Relative score as a function of lag distance within cont-ret pairs. Examining the effect of temporal distance during encoding of cont-ret pairs on relative score, we found a significant main effect of distance (*p* < 0.05, BF_10_ = 3.68) such that pairs that were closer exhibited higher scores than distant pairs. The post hoc comparison between short and long lags was significant (*p*_*bonf*_ < *0*.042, BF_10_ = 3.7). Error bars denote SEM.

The mean relative score of short lags was the highest (mean = 0.74, SEM = 0.03), followed by medium lags (mean = 0.66, SEM = 0.04), and finally long lags (mean = 0.57, SEM = 0.05). We found a significant main effect of lag (*F*_2,42_ = 4.46, *p* < 0.05, η²_p_ = 0.175; see Fig. 3). A Bayesian RMANOVA supported this finding, with moderate evidence (BF_10_ = 3.68) in comparison to a null model including only experiment number as a nuisance variable. A post hoc comparison found a significant difference after Bonferroni correction only for the difference between short and long lags (*t*_(21)_ = 2.67; *p*_*bonf*_ = *0*.042), with substantial support from Bayesian analysis with a BF_10_ (uncorrected) of 3.7. Importantly, these finding are in stark contrast to the prediction derived from Distinctiveness Approach that the longest lags will be the most temporally distinct and hence have the highest relative score.

### Prediction 3: Relative order as a function of distance score across pair categories

We examined relative score as a function of the distance score, across pair categories. According to the Temporal Distinctiveness Approach, we would expect that pairs with greater distance scores, reflecting greater perceived distance than the actual distance in encoding, would show enhanced relative order in comparison to pairs with smaller perceived distance. In contrast, the Shared Context Approach predicts the opposite: increased relative order for items with small distance scores. For each subject we performed a median split of the pairs according to their distance score into two bins (short/long distance). We included only subjects who had at least three pairs from each bin. Due to the relatively restricted range of distance scores, we did not split the pairs into terciles as done in Prediction 2 because this resulted in fewer than ten subjects that had enough observations per bin. To increase statistical power, we combined data from both experiments, and included experiment type as a covariate within the RMANOVA that was performed with distance score as the independent variable, and relative score as the dependent variable.

Pairs with small distance scores exhibited a higher relative score (mean = 0.76, SEM = 0.01) than pairs with large distance scores (mean = 0.59, SEM = 0.02). We found a significant main effect of distance (*F*_1,52_ = 42.20, *p* < 0.001, η²_p_ = 0.44). A Bayesian RMANOVA including experiment type as a nuisance variable, found extremely strong evidence for the model including distance in comparison to the null model (BF_10_ = 4.28*10^7^). These findings further support the Shared Context Approach that emphasizes perceived proximity in supporting relative order.

### Prediction 4: Equivalent absolute order for pairs encoded and retrieved contiguously (cont-enc-ret) over pairs not encoded contiguously (cont-ret)

In order to support our prediction that there is no difference in the absolute score of cont-enc-ret and cont-ret pairs, we used Bayesian statistics that are capable of providing evidence in favor of a null model^34,35^. To test this hypothesis, we only examined the absolute score of the first item that was ordered, because the second item was typically biased in its ordering by the placement of the first (see Supplemental Material Section D for further discussion). In the first experiment, the mean absolute score for cont-enc-ret pairs was 0.882 (SEM = 0.01) and for cont-ret pairs was 0.884 (SEM = 0.01). A Bayesian paired t-test found moderate support for the null hypothesis (BF_01_ = 5.04). We further examined the possibility of a testing effect confound on absolute order, similar to Control Analysis 1 A, and performed a similar analysis for absolute order and found no evidence for such an interaction (see Supplemental Material Section B). Also, noting that the scores are close to the maximum value of one and to account for possible ceiling effects, we also examined the absolute deviation that is the unsigned distance between the ordering of an item and its initial position. Importantly, these scores are not close to ceiling (or floor). The results showed a similar pattern to those of the absolute scores. The absolute deviation of cont-enc-ret pairs was 1.27 (SEM = 0.1) and of cont-ret pairs was 1.3 (SEM = 0.09). A Bayesian paired t-test found moderate support for the null hypothesis (BF_01_ = 4.95). Experiment 2 replicated these results. Cont-enc-ret pairs had a mean absolute score of 0.87 (SEM = 0.01), cont-ret had a mean absolute score of 0.86 (SEM = 0.02), and cont-enc had a mean absolute score of 0.87 (SEM = 0.02). A Bayesian one-way RMANOVA of absolute score yielded moderate evidence in favor of the null hypothesis, that there is no difference between the pair types (BF_01_ = 7.2). Examining absolute deviation yielded similar results (BF_01_ = 4.8). Confirming our hypothesis that there is no difference between the absolute sequence memory of contiguously and non-contiguously encoded items.

### Prediction 5: Greater correlation for relative score and TCE magnitude in comparison to absolute score and TCE magnitude

**Figure 4:**
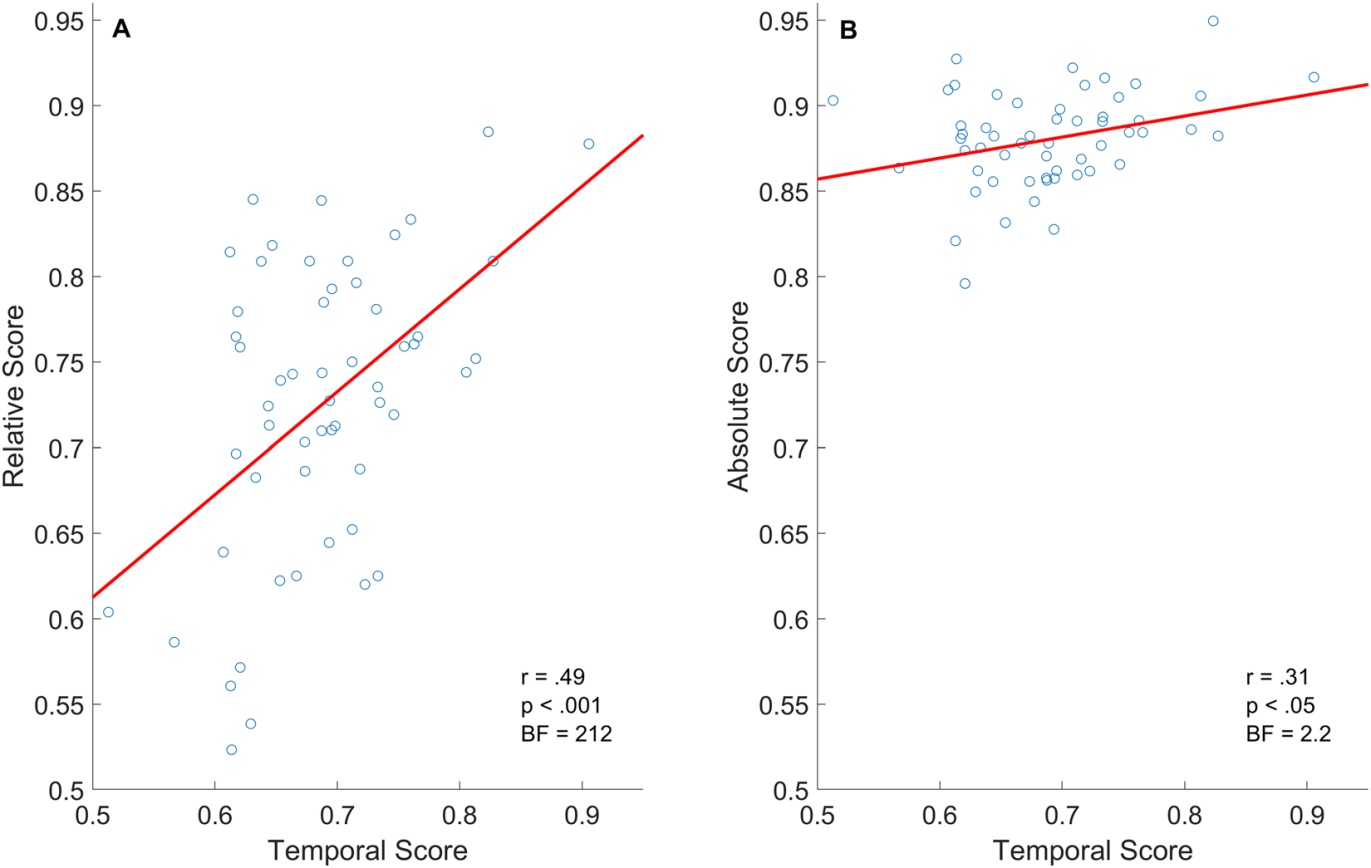
Across subject correlations of temporal score with relative score (Panel A) and of temporal score with absolute score (Panel B). In comparison to the correlation of absolute score, the correlation of relative score with temporal score was larger and had substantial Bayesian support.

To further probe the dissociation between relative and absolute scores we examined their relation to TCE magnitude. TCE was indexed by the temporal score^12^, a measure of utilization of temporal context during free recall (see Methods for description of measure). We examined the across subject correlations between temporal scores and relative scores, as well as between temporal scores and absolute scores. To increase statistical power, we combined subjects from both experiments giving us a sample of n = 55, and calculated relative and absolute scores collapsed across pair categories. Both the correlation between relative and temporal scores (r_p_ = 0.49, *p* < 0.001), and absolute and temporal score (r_p_ = 0.31, *p* < 0.05) were significant with the former being greater (see Fig. 4). To ensure that the correlation between absolute and temporal score was not reduced because of absolute scores’ smaller range of values, we also examined the correlation between absolute deviation and temporal score which showed the same trend (r_p_ = −0.34, *p* < 0.05). To tease apart the relation between absolute and relative order to temporal score, we examined their partial correlations. We found that the partial correlation between relative and temporal scores while controlling for absolute order was significant (*r* = 0.43, *p* = 0.001). In contrast, the partial correlation between absolute and temporal scores when controlling for relative order was not significant (*r* = 0.18, *p* = 0.2). Bayesian correlation analysis found that the evidence supporting correlation between relative and temporal score was extremely strong BF_10_ = 212, whereas the evidence for the correlation of absolute score was anecdotal BF_10_ = 2.23, and absolute deviation was moderate BF_10_ = 3.7. These results further support the dissociation between absolute and relative order, with the latter being more strongly related to utilization of temporal context during free recall. Finally, to further probe the dissociation between absolute and relative score, we also examined the across subject correlation to distance score during the ordering task, that reflects the perceived distance between the items. Again, we found the expected trend that relative order was more strongly associated with accurate distance scores as opposed to absolute, yet these results were not corroborated by Bayesian analysis so we interpret them with caution and report them fully in the Supplemental Material Section E.

### Prediction 6: Equivalent relative score for cont-enc-ret and cont-enc: The contribution of encoding as opposed to recall on relative score

To tease apart the contribution of encoding and recall processes to memory of relative order, in Experiment 2 we introduced an additional pair category, cont-enc. To briefly reiterate, the cont-enc category includes pairs of items that were encoded contiguously and both items were recalled yet not contiguously. Importantly, they differ from cont-enc-ret only with regards to the recall phase, allowing us to dissociate encoding and recall processes. In Experiment 2 the mean relative score of cont-enc-ret was 0.80 (SEM = 0.02), and the mean relative score of cont-enc was 0.77 (SEM = 0.026). Post hoc comparisons, with Bonferroni correction, of the ANOVA described in Prediction 1 found that that there was no significant difference between the two (*t*_(24)_ = 0.83, *p*_*bonf*_  > 0.9). Importantly, the equivalent Bayesian analysis yielded moderate evidence in favor of the null model (BF_01_ = 5.91) that there is no difference between them. The lack of difference between the two categories implicates encoding process in the retention of explicit relative order.

### Prediction 7: Improved distance score for cont-enc-ret pairs in comparison to cont-enc pairs

To test our prediction that cont-enc-ret pairs are the basis for formation of temporally structured episodes, we compared their distance score to cont-enc pairs’ score. To reiterate, a distance score is the difference between the distance between the items in the ordering phase and their true distance during the study phase. Note, that the comparison between cont-enc-ret and cont-ret pairs is not valid since the latter’s range of distance scores include both positive values (i.e. distancing of pair members) and negative values (i.e. bringing them closer). Therefore, we only compared cont-enc-ret to cont-enc—pairs that were studied contiguously but not recalled successively. Both pair categories have a range of only positive values, because it is not possible to order them closer to each other than their actual distance (i.e. zero distance). In line with our prediction, cont-enc-ret pairs were ordered closer to each other than cont-enc pairs, despite their true distance being the same. Thus, cont-enc-ret pairs had a significantly smaller mean distance score than cont-enc pairs (Mean cont-enc-ret = 0.4, SEM = 0.1; Mean cont-enc = 0.78, SEM = 0.09; *t*_(24)_ = −4.17, *p* < 0.001, Cohen’s *d* = *−*0.83). The equivalent Bayesian analysis yielded strong evidence in favor of H_1_ (BF_10_ = 89.5), that cont-enc-ret pairs are ordered closer to each other reflecting their binding into a temporally-structured episode.

Further supporting our prediction that cont-enc-ret pairs constitute a temporally-structured episode, we examined the proportion of pairs in each category that had both a perfect relative score and a perfect distance score (i.e, score of zero). Intuitively, this analysis compares the pairs’ tendency to ‘stick together’ in the correct order. Using a paired t-test to compare the proportion of pairs that have both perfect distance and relative scores, we found a statistically significant difference (*t*_(24)_ = 3.02, *p* < *0*.01, Cohen’s *d* = 0.6) between cont-enc-ret pairs (Mean = 71%, SEM = 5%) and cont-enc pairs (Mean = 57%, SEM = 4%). The equivalent Bayesian analysis yielded moderate evidence (BF_10_ = 7.4) in favor of the difference. These results corroborate our hypothesis that cont-enc-ret pairs are bound together into a temporally-coherent episode, whose items are explicitly ordered relative to one another.

## Discussion

The current study examined how internally-driven temporal context, as manifested by the TCE, affects explicit sequence memory. Three main lines of results converge on the role of internally-driven temporal context in the formation of temporally-structured episodes. These results, discussed in detail below, are: (1) The contribution of temporal proximity to retention of relative order, as predicted by the Shared Context Approach as opposed to the Temporal Distinctiveness Approach. (2) A dissociation between relative and absolute order. (3) The contribution of contiguous encoding as opposed to contiguous retrieval in supporting relative order.

First, three findings support the role of temporal proximity and shared context in enhancing relative order. In line with the Shared Context approach, relative order was enhanced for items encoded contiguously (cont-enc-ret & cont-enc) compared to items encoded non-contiguously (cont-ret; Prediction 1). Even within items encoded non-contiguously (cont-ret), the proximity of items during encoding was predictive of enhanced relative order (Prediction 2). Finally, items that were perceived as proximal (i.e., small distance scores) exhibited superior relative order over items perceived as more distal (i.e., large distance scores; Prediction 3). Contrasting the predictions of the two major approaches, the Shared Context as opposed to the Temporal Distinctiveness, the current study found strong support for the former. This finding is novel and contrasts a wealth of prior findings supporting temporal distance and distinctiveness in driving relative order^13,18,19^. The support for the Shared Context Approach is in line with the idea that this approach provides a better account in cases in which encoding relies on relational and contextual processes. We used a free recall task that is an internally-driven memory search process, in which performance strongly relies on shared temporal context and inter-item associations^12,36,37^ (though see^38–40^ for cases in which free recall relies less on such processes). In contrast, when the task is markedly item-focused, the Distinctiveness Approach trumps the Shared Context Approach^8^. This was the case in a recent study^41^, which found that distinctiveness enhances item’s source memory of color. These diverging findings emphasize the importance of task demands in determining the reliance on shared context or temporal distinctiveness in supporting sequence memory.

Second, while relative order differed between contiguous and non-contiguous encoding (cont-enc-ret & cont-enc versus cont-ret categories), absolute order did not differ between the categories. This finding was replicated across both experiments (Predictions 1 and 4). Further supporting the dissociation between the two types of order, across-subject correlations between the utilization of temporal context during recall (i.e. Temporal Score) were stronger for relative order as opposed to absolute order (Prediction 5). This dissociation is in line with neuroimaging evidence that has found distinct networks supporting each of the two types of order^17,26^. Absolute order is supported by frontal networks, whereas relative order is supported by medial temporal networks that are also implicated in the TCE^28,29^. Likewise, hierarchal models of memory^22^ posit that within clusters recall proceeds in a serial manner relying on the items’ temporal order and the relation between them. This results in an enhancement of relative order memory for items within the same cluster as compared to items from different clusters, but it does not affect absolute order memory for any of the items.

Finally, comparing the contribution of encoding as opposed to retrieval processes, we found that pairs only encoded contiguously (cont-enc) and pairs both encoded and retrieved contiguously (cont-enc-ret) showed equivalent levels of relative order (Prediction 6). In addition, both cont-enc-ret and cont-enc pairs showed higher levels of relative order in comparison to pairs that were not encoded contiguously (cont-ret). Thus, contiguous encoding is presumably the driving force behind enhanced relative order. This finding is in line with work which found that manipulations at encoding (i.e., longer presentation rates and instructions emphasizing relational processing) promoted the reliance on shared context and the formation of episodes^8^. Further highlighting the importance of encoding processes, studies have shown that incidental encoding significantly reduces the TCE^42^, and manipulation of the encoding task affects the magnitude of the TCE^43^. Finally, divided attention during encoding, as opposed to retrieval, greatly impairs recall^24,25^. Despite the emphasis on contiguity at encoding, it should be noted that contiguous encoding and retrieval enhanced the perceived proximity between items (Prediction 7), as would be expected for items from the same episode^30^. The role of retrieval in the formation of episodes may be through the reinstatement of items recalled which further augments their association with each other via a shared context at retrieval in addition to encoding. Thus, items that are contiguous at both encoding *and* retrieval are more likely to share internally-driven contexts, as compared to items that are contiguous only at encoding. Importantly, in the current study all categories included items which were successfully recalled. To further examine the role of retrieval, future studies should also compare sequence memory of items not recalled.

The current findings provide an important extension to previous research that has examined the phenomenological experience associated with contiguous versus non-contiguous recalls. Temporally-contiguous recalls are more likely to be judged with higher confidence ratings^44^, and to be experienced as ‘recollected’ as opposed to ‘familiar’ (a finding obtained using the ‘Remember/Know’ paradigm^39,40^). Given the everyday importance of recalling the order in which events occurred, our current findings that contiguously-encoded recalls exhibit superior explicit sequence memory further support the debated ecological validity of the TCE^42,45^, by positing it a role in the retention of sequential information for temporally contiguous items. Future research should examine whether the subjective strength and vividness experienced when recalling temporally-contiguous items is derived from explicit recollection of the items’ order.

To conclude, the current study provides novel converging evidence concerning the relation between internally-driven temporal context and explicit sequence memory. Shared temporal context between proximal items, as captured by the TCE, gives rise to explicit memory for the sequence of items within an episode, specifically their relative order and perceived distance and not their absolute order. Furthermore, the formation of episodes seems to rely more on contiguous encoding as opposed to retrieval. This provides novel evidence in support of the crucial role of internally-driven temporal context in creating a scaffold on which items are bound together to form a temporally coherent episode that includes explicit sequence memory.

## Methods

### Participants

#### Experiment 1

Participants were 30 Ben-Gurion University native Hebrew speaking students (25 women) aged 21–27 years (mean 23.5). All participants passed the a priori exclusion criterion of having at least 5 observations in each of the order categories, ensuring sufficient observations for statistical analyses. All experimental procedures were approved by the Ben-Gurion University ethics committee. The study was carried out in accordance with the relevant guidelines and regulations. All participants provided informed written consent to participate in the study, and were paid or given course credit as reimbursement for their participation.

#### Experiment 2

Participants were 39 native Hebrew speaking Ben-Gurion University students, of these three were excluded due to technical failures and another 11 were excluded due to having fewer than 5 pairs in one of the pair categories. This resulted in a total of 25 participants (18 women) aged 23–26 years (mean 24.12 years). The high exclusion rate was a result of the difficulty in finding sufficient pairs for each category because the inclusion of items in one category excludes them from being in a second (see Supplemental Material Table 1 for the number of pairs presented per condition).

## Materials

Materials were identical for both experiments. Stimuli consisted of 661 Hebrew nouns, 3–6 letters long (mean word length = 4.21 letters). For each participant, 30 lists of 16 words each were randomly sampled without replacement from this pool of words. Of the 30 lists, two served as practice lists (see Procedure below) and 28 as test lists. Stimuli were presented and responses were recorded with the Expyriment program^46^.

## Procedure

### Experiment 1

The experiment consisted of two conditions. The first was a free recall task without an ordering task (no-order condition) that included one practice list and 9 test lists. The second condition was a free recall task in which recall of each list was followed by an ordering task (order condition). This condition consisted of one practice list and 19 test lists. The ‘no-order condition’ always preceded the order condition. At the start of each condition, participants were presented with detailed instructions and performed a single practice list that was discarded from the analysis.

For each list in the free recall task, participants were presented with words that appeared on the center of the screen for 2 s, followed by a fixation point that appeared for 1 s. After the presentation of the entire list, a text box appeared on the screen and participants were instructed to accurately type as many words as they could recall. After each recalled word, participants pressed Enter and the box was cleared. Importantly, participants were instructed to recall as many words as they could remember regardless of their order. The recall phase lasted 60 s, after which they could rest and start the presentation of the next list by pressing a key (see Fig. 1).

In the order condition, following recall as described in the no-order condition, participants performed an ordering task. In each ordering trial, they were presented with a pair of words that they had recalled. The word pair appeared on the top of the screen, and on the bottom there were 16 empty slots (see Fig. 1). Participants were instructed to order both of the words, using the mouse, according to their initial position during the study phase. Each ordering task lasted 13 s, after which a new pair appeared and the slots at the bottom of the screen were refreshed. During the 13 s, participants were able to change the order of the items as many times as they wanted.

The pairs presented in the order phase were classified into four categories, the cont-enc category was only used in the second experiment. To illustrate, if a participant recalled the following items (labeled according to their serial position during the study phase): 16, 15, 14, 12, 4, 7, 8, 2, 10, 13; the four categories were: (1) Pairs encoded and retrieved contiguously, coined ‘cont-enc-ret’. For example, the pair 7, 8. (2) Pairs retrieved but not encoded contiguously, coined ‘cont-ret’. For example, the pair 2, 10. (3) Pairs encoded but not retrieved contiguously, coined ‘cont-enc’. For example, 12, 13. (4) A pair of items from the first three items recalled, regardless of their lag. For example, 16, 15. These items might reflect retrieval from a short term memory (STM) store^47^ (but see^48^ for contrary view). Therefore, the results reported here do not include pairs from this category. Still, additional analyses revealed that combining this category within the categories above generally yielded very similar results that are reported in the Supplemental Material Section G. Importantly, no item was presented twice, even if it could be classified into more than one category. The sequence of pairs and the position of the words within a pair (i.e., if the word appeared on the right or left side of the screen) was randomly determined. Because the number of pairs from each category is dependent on the participant’s recall, we developed an algorithm that extracted pairs in an optimal manner ensuring that the number of pairs in each category was maximal, and that the number of pairs was similar across categories. At the end of the experiment participants completed the “Dissociative Experience Scale” questionnaire^49^. The purpose of this questionnaire was extraneous to the current endeavor and, therefore, its results will not be discussed here.

### Procedure: experiment 2

The procedure was identical to Experiment 1 except for two differences. First, the order and no-order lists were randomly intermixed throughout the experiment. Accordingly, at the beginning of the experiment, participants performed two practice lists: the first without order and the second with. These lists were discarded from the analysis. Second, in addition to cont-enc-ret and cont-ret categories, we also included the cont-enc category described above. Due to the constraint that each item is presented once during the ordering phase, cont-enc were not presented in the previous experiment in order to ensure that a sufficient number of observations from the other categories were obtained (see Supplemental Table 1 for distribution of number of pairs across conditions).

## Data Analysis

All data were processed with in-house Matlab scripts (The Mathworks, Natick, MA, USA) and statistical analyses were performed with JASP version 0.9^50^. Bayesian analyses were performed with the default priors and Cauchy width provided by JASP.

## Measures

### Recall measures

For the recall phase two measures were obtained. First, the mean number of correct items recalled per list. Second, utilization of temporal context was measured using the ‘temporal score’^12^. Briefly stated, the temporal score is a single value signifying a subject’s reliance on temporal context. The score ranges from one to zero, one reflects ‘optimal’ utilization of temporal context and a score of 0.5 reflects chance performance (see Supplemental Material Section F for details concerning other recall performance measures).

### Order measures

#### Relative score

A measure of each pair’s relative order, regardless of the placement of the items’ absolute order. The score is either one for correct relative placement of pair members or zero for incorrect placement of pair members. For example, if items, 7 and 8 were respectively placed in the positions 1 and 16 or in positions 2 and 3 both orderings would result in a relative score of 1.

#### Absolute score

A measure of each item’s absolute order. This measure is based on the absolute deviation between the item’s ordered position and its true position. Importantly, absolute score is only based on the magnitude of the deviation from the true position, regardless of whether the item was ordered before or after its true position. We controlled for a potential confound concerning the dependence of the range of the absolute deviations on the items’ serial position. That is, items from the beginning or end of the list have a larger range of possible deviation values over items from the middle of the list. To account for this, each absolute deviation was divided by the maximal possible deviation. The absolute score was calculated as $1-\frac{{\rm{actual}}\,{\rm{deviation}}}{\max \,{\rm{deviation}}},$ ranging from a value of one for perfect placement and zero for the largest possible deviation that could be made given the item’s actual position. In contrast to the relative score that is scored per pair, the absolute score is calculated separately for each item. The analysis reported focused on the ordering of the first item in each pair because cont-ret pair members generally exhibit a strong negative distance score, reflecting the tendency to order the second item closer to the first, thus confounding explicit knowledge for absolute order of the second item (see Supplemental Material Section D).

#### Distance score

A measure of whether the items were placed closer or farther apart in relation to their distance from one another during study. This score was calculated by subtracting the distance between the items that were ordered from their distance during the study phase. Thus, negative values signify that items were placed closer to each other than they had appeared at study, and positive values signify that they were placed farther apart. A score of zero indicates that the items were placed in their correct distance. Importantly, this measure is independent of the previous measures of relative and absolute order.

## Supplementary information

Supplemental Information.

## Data Availability

Data is available at https://data.mendeley.com/datasets/pkf67bxzhy/draft?a = 0f2e610a-0b73-4436-b3a3-6e4f6248c640
