# Supplementary material for: Explicit Sequence Memory in Recall of Temporally-structured Episodes
Source: Sci Rep. 2020 Feb 14;10:2666. doi: 10.1038/s41598-020-59472-8 (PMC7021684; doi:10.1038/s41598-020-59472-8)
Supplement: Supplementary file 1 — Supplemental Information. [file 41598_2020_59472_MOESM1_ESM.pdf]

Supplemental Information for:

Explicit Sequence Memory in Recall of Temporally-structured Episodes

Yonatan Stern, Ron Katz and Talya Sadeh

Supplemental Section A: Confound of testing effect on relative score difference

An alternative explanation for the increase in the cont-enc-ret relative score in comparison to cont-ret is that participants were not relying on the pairs' position during the study phase but rather were ordering them according to their relative order during recall. This can be construed as a testing effect, whereby the recollection of the pair creates an enhanced memory trace of the testing phase [2]. Thus, it may be that cont-enc-ret shows enhanced relative order because participants are simply ordering according to their recall creating an advantage for pairs with forward transitions (i.e. a positive lags). Such forward transitions are more abundant in cont-enc-ret than cont-ret category—a finding known as 'forward asymmetry' in the TCE[3]. That is, lag +1 are more likely than lag -1 recalls, whereas for greater lags positive and negative transitions are generally equally probable. Therefore, the higher relative score for cont-enc-ret compared to cont-ret may be a result of the forward asymmetry effect that results in a higher proportion of forward transitions in cont-enc-ret.

In order to examine this possibility, we conducted a two-way RMANOVA with lag direction (forward/ backward) and pair category (cont-enc-ret/ cont-ret) as independent variables and relative score as the dependent variable. To increase statistical power we combined data from both experiments, and included in the analysis only participants who had at least 3 pairs in each of the four cells in order to ensure that values were based on sufficient observations (N=41). An RMANOVA with experiment number as a covariate found a significant main effect of pair category ($F_{1,39} = 29.9$, $p < .001$, $\eta^2_p = 0.43$), signifying a greater relative score for cont-enc-ret (mean = 0.80, SEM = 0.02) over cont-ret (mean = 0.65, SEM = 0.03). The direction main effect was also significant ($F_{1,39} = 11.1$, $p = .002$, $\eta^2_p = 0.22$), with forward transitions having a higher relative score (mean = 0.76, SEM = 0.03) than backward transitions (mean = 0.68, SEM = 0.03), presumably reflecting the testing effect. Importantly, the interaction between pair type and direction was not significant ($F_{1,39} < 1$, $p > .8$), indicating that the testing effect and the advantage of forward transitions did not differ between cont-enc-ret and cont-ret pairs (see Figure 2 in main text). This was further corroborated by examining the simple effects of pair category as a function of direction. In both cases, cont-enc-ret's relative order was higher than cont-ret's (Simple effect of Forward, $F_{1,39} = 18.5$, $p < .001$; Backward, $F_{1,39} = 14.8$, $p < .001$). Accordingly, cont-enc-ret's increased accuracy cannot be solely explained by the testing effect, as is evident

from the significant increase even in backward transitions where the testing effect is absent. Furthermore, the non-significant interaction of pair type and transition direction indicates that although the testing effect increases the accuracy of forward transitions, it acts in a similar manner on both cont-enc-ret and cont-ret. The importance of pair type to explaining cont-enc-ret's superior relative order is further corroborated by the Bayes factor of inclusion ($BF_{Inclusion}$). Briefly stated, $BF_{Inclusion}$ is a measure of the evidence supporting the inclusion of a given factor that is obtained by comparing the model with the factor to a similar class of models without the factor [4]. We found that the $BF_{Inclusion}$ for lag type was extremely strong ($BF_{Inclusion} = 1*10^6$), in comparison to direction ($BF_{Inclusion} = 26$), and pertinently there was moderate evidence in favor of not including the interaction term ($BF_{Inclusion} = 4.05$). Together, these analyses support our claim that although there is a significant effect of transition direction and testing, pair category is an independent strong effect that explains considerably more of the variance in the relative order measure. Similar analyses using absolute score and distance score as the dependent variable were also performed, in order to examine the contribution of testing effects to their findings, yet no interaction with pair category was found (see Supplemental Material Section B), further supporting our claim that these effects are also not driven by retrieval related testing effects.

Supplemental Section B: Testing effects and findings of absolute score and distance score

In order to refute the potential confound of testing effects on the findings concerning absolute score and distance score, we performed analyses analogous to Supplemental analysis 1B and Supplemental Section A that were performed on relative score.

We examined the effect of direction on absolute order via a RMANOVA that included both pair category (cont-enc-ret/ cont-ret) and transition direction (forward/ backward) as factors. To increase statistical power we combined data from both experiments, and included in the analysis only participants who had at least 3 pairs in each of the four cells in order to ensure that values were based on sufficient observations (N=41). Experiment number was included as a covariate. The interaction between the two factors was not significant ($F_{1,40} < 1$ $p = .7$). A Bayesian RMNANOVA revealed that the best model was that that included both pair category and transition direction, and that is was moderately better than a model including also the interaction between pair category and transition direction ($BF_{10} = 5.43$). Thus, testing effects and

the direction of recall cannot explain the finding of no difference in absolute score across categories.

Next, we examined the effects of direction on distance score via a RMANOVA that included both pair category (cont-enc-ret/ cont-enc) and transition direction (forward/backward) as independent variables. This analysis included subjects only from the second experiment where the pair category cont-enc was included. The interaction between the two factors was not significant ($F_{1,17} = 1.7$ $p = .21$). Bayesian analyses did not provide evidence for the inclusion or exclusion of the interaction term ($BF_{Inclusion} = 1.23$). That is comparing a model that included both transition direction, pair category and their interaction was only anecdotally better than a model including transition direction and pair category. Furthermore, the evidence for inclusion of transition direction ($BF_{Inclusion} = 1.26$) was anecdotal and several magnitudes smaller than support for pair category ($BF_{Inclusion} = 4*10^5$). Together these results support our claim that transition direction and testing effects cannot explain the difference we found in distance scores between cont-enc-ret and cont-enc.

Supplemental Section C: Confound of condition type on relative score difference

A potential confound of the results in Prediction 1 is that due to the inclusion of an ordering task following recall, recall was not performed 'freely' and was affected by the succeeding ordering task. In the first experiment, the 'no order' condition always preceded the 'order' condition. Subjects during the 'no-order' condition were not aware of the succeeding 'order' condition, thus allowing us to obtain 'pure' free recall measures from the first condition. Therefore, in subsequent order analyses, we included as covariates the difference in recall measures (mean number of items recalled and temporal score) between conditions.

In line with previous work by Mulligan and Lozito (2007[5]) that compared standard recall performance with recall performance that was intermixed with an order reconstruction task (see especially experiments 2a & 2b), we found that the order condition slightly enhanced overall recall performance and enhanced the utilization of temporal context. In the order condition, the mean number of items recalled per list was 8.96 (SEM = 0.35), whereas in the no-order condition it was 8.55 (SEM = 0.32). This difference approached significance ($t_{29} = -1.95$; $p = .06$; Cohen's

$d = 0.36$). The equivalent Bayesian analysis did not provide evidence in favor of either hypothesis ($BF_{10} = 1.02$). Comparing the temporal score that examines the probability of making a transition to items studied nearby, and is measure of temporal context utilization[6] (see Methods section for further details), the order condition had a mean of 0.67 (SEM = 0.013), whereas the no-order condition had a mean of 0.62 (SEM = 0.014). This difference was significant ($t_{29} = 3.32$; $p < .005$; Cohen's $d = 0.61$), and the equivalent Bayesian analysis provided strong evidence ($BF_{10} = 15.24$) in favor of the hypothesis positing a difference between the conditions. The significant differences between conditions may be due either to enhanced attention to order stemming from the ordering task or simply due to changes in time on task (see Supplemental Figures 1 & 2 for additional differences in memory performance measures between the two conditions).

Importantly, we were interested in examining whether differences between conditions in recall measures could account for our finding regarding relative order. Accordingly, we performed a one-way repeated measures ANOVA with pair category as an independent variable, relative score as the dependent variable, and included as covariates the differences between the two list conditions in overall recall performance and temporal score. This revealed a significant effect of pair category on relative score ($F_{1,29} = 18.89$; $p < .001$; $\eta^2_p = 0.42$). Thus supporting our claim that differences in relative order cannot be explained by changes in recall performance or utilization of temporal context due to the differences between list conditions such as experimental demand or time on task. Yet also the interaction between pair category and the covariate temporal score was marginally significant ($F_{1,29} = 4.5$; $p = .04$; $\eta^2_p = 0.14$), potentially complicating the main effect of interest that we found, if the covariate and dependent variable are intrinsically linked to each other[7]. In other words, one could argue that the difference in relative order between cont-enc-ret and cont- ret is driven by enhanced attention to order during the order condition that led participants to order cont-enc-ret pairs with greater accuracy. Accordingly, the main effect of pair type on relative score is merely an artifact of the experimental design. However, this interpretation seems highly unlikely for three reasons. First, there is no significant correlation between the difference in temporal score and the difference in the relative score of cont-enc-ret and cont-ret ($r_p = .28$, $p > .12$). Such a correlation would have been expected if cont-enc-ret 's advantage arose from enhanced attention to order during the order condition. Second, the effect size of pair category ($\eta^2_p = 0.42$) is considerably larger than the interaction's effect size

(η^2_p = 0.14), supporting our claim that the difference between conditions explains only a small proportion of the variance in comparison to the main effect of pair category. Finally and most importantly, we conducted a Bayesian RMANOVA with pair category as an independent variable, relative score as the dependent variable, and the differences between the two list conditions in overall recall performance and temporal score included as nuisance variables within the null model. This analysis revealed extremely strong evidence in favor of the model including the main effect of pair category with a Bayes Factor (BF_{10}) of > 21,000 in comparison to the null model. Thus, in line with our prediction, contiguous recalls (cont-enc-ret) exhibit superior explicit relative order over non-contiguous recalls (e.g cont-ret), even when accounting for differences between the conditions. Moreover, the difference between conditions is not intrinsically related to the order scores.

In the second experiment we followed a more common method of intermixing no-order and order lists [5,8], in order to deal with the confound of condition order. In contrast to the first experiment, participants recalled the same number of items in each type of lists, a mean of 9.22 items per list (SEM = 0.41) in the order lists and a mean of 9.22 items per list (SEM = 0.38) in the no-order lists. Comparing the utilization of temporal context as measured by the temporal score, the order lists had a mean temporal score of 0.71 (SEM = 0.014) whereas the no order lists had a mean of 0.69 (SEM = 0.018). This difference was not significant ($t_{(24)}$ = -1.8, p = .08). The equivalent Bayesian analysis resulted in a BF_{01} of 1.16, not providing evidence in favor of either hypothesis. These findings show that the intermixing of the lists reduced the differences in performance between the two types of lists. Importantly, as noted above, the difference in relative score between cont-enc-ret and cont-ret was replicated—a finding arguing against the possibility that the difference stems from a unique recall strategy adopted during the order condition.

Supplemental Section D: Justification for examining absolute score of the first ordered item

In order to test our hypothesis that there is no difference between the absolute score of cont-enc-ret and cont-ret, we compared the absolute scores of only the first item ordered in each pair type. The justification for this lies in our finding that cont-ret pairs exhibit a strong negative

distance score, presumably reflecting the tendency to order the second item closer to the first. This, in turn, results in a low absolute score for the second cont-ret item. Thus, in the first experiment, the absolute score of the first cont-ret item ordered (mean = 0.88, SEM = 0.01) was greater than the second item ordered (mean = 0.79, SEM = 0.02). We used the Wilcoxon signed-rank to test the significance of this difference ($W = 446$, $p < .001$, $r_{pb} = 0.92$), because the assumption of normality was not met with the Shapiro-Wilk test ($W = 0.82$, $p < .001$). This result was further corroborated with the equivalent Bayesian test that found extremely strong evidence in favor of the difference ($B_{10} = 487$). We argue that the lower absolute score of the second item is a result of it being placed closer to the first item, as revealed in our distance measures. These results thus justify our choice to examine absolute order only for the first item.

Supplemental Material Section E: Across-subject Correlation between Distance, Relative and Absolute Scores

To further probe the dissociation between absolute and relative score, we also examined the across subject correlation to distance score during the ordering task. In line with Prediction 5, we hypothesized that relative score would be more strongly correlated with distance score than with absolute score. To reiterate, distance score reflects the perceived distance during ordering between the items in comparison to their true distance during encoding, with a score of zero signifying that items were placed with their correct distance. We examined the correlation of the absolute value of distance score, thus equating positive (i.e. placing items farther) and negative (i.e. placing items closer) distance score errors. This transformation is necessary because we were interested in examining the correlation between distance score’s accuracy (i.e. a score of zero) and relative/absolute score. Without the transformation if, for example, a positive correlation was found between distance and absolute order it would signify that subjects that misplaced pairs closer to each other (i.e. a negative distance score) had low absolute scores whereas subjects that misplaced pairs farther apart (i.e. positive distance scores) had higher absolute scores, and most importantly that subjects that placed items correctly (i.e. a distance score of zero) had average absolute scores. Thus, without the transformation, the correlation captures the relation to different types of distance score errors rather than to distance score accuracy. In line with the dissociation between relative and absolute order we found that relative

score was significantly and negatively correlated with distance score errors ($r_p = -.29$, $p < .05$), whereas absolute score was not significantly correlated with distance score errors ($r_p = -.17$, $p = .2$). Equivalent Bayesian analyses only provided anecdotal corroboration, with a BF_{10} of 1.63 supporting the association with relative score, and a BF_{01} of 2.6 supporting the lack of association between absolute order and distance score.

Supplemental Material Section F: Free Recall Measures & Distribution of Pair Categories

In order to further examine differences between the two list conditions (order/no-order) in Experiment 1, we include below the serial position curve (SPC) of the two list conditions, and the lag-CRP. Finally, we include the distribution of the number of pairs from each category that the order measures were based upon.

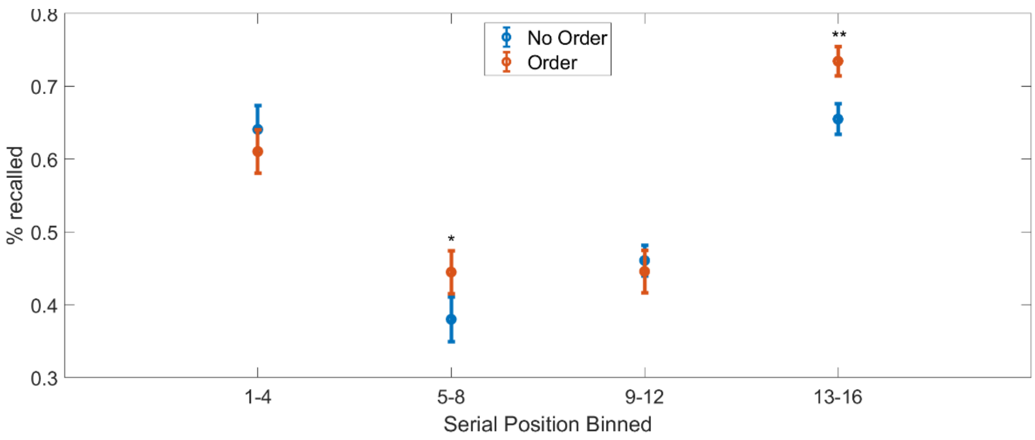

Supplemental Figure 1. Serial position curve from Experiment 1, split across the two conditions types 'order' & 'no order'. Error bars are SEM. * = $p_{bonf} < .05$, ** = $p_{bonf} < .001$.

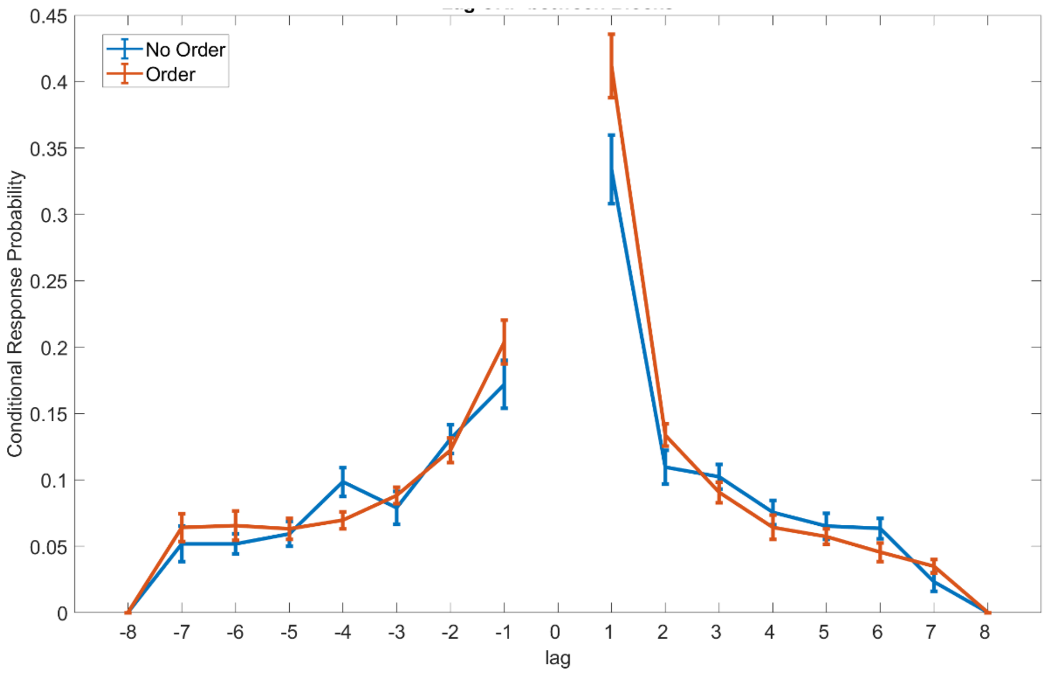

Supplemental Figure 2. Lag-CRP between the two list conditions (order/no-order) in Experiment 1. Lag-CRP is the conditional probability of transitioning to items at different lags during recall[3]. None of the differences reached statistical significance after Bonferroni correction for multiple comparisons.

Experiment	Pair Type	Mean number of items for condition	SD	Range
1	cont-enc-ret	36	19.4	7, 82
	cont-ret	16.6	7.4	5, 33
2	cont-enc-ret	19.6	10.6	7, 61
	cont -ret	10	3	5, 15
	cont-enc	15.8	7.9	5, 36

Supplemental Table 1. Distribution of pair categories across both experiments. Participants were not included in experiment if they had fewer than 5 items per category.

Supplemental Section G: Analyses including Short-Term Memory (STM) items

In the analyses reported in the manuscript, we excluded the first three items recalled because according to influential models of memory, they reflect retrieval from a short term memory store[1]. In this section we report the results of the major analyses that appear in the manuscript, this time including all items. The results show the same general pattern as that found in the data excluding the first three items.

Superior relative order for pairs encoded and retrieved contiguously (cont-enc-ret) over pairs not encoded contiguously (cont-ret)

In both experiments we found strong evidence for enhanced relative order for cont-enc-ret pairs in comparison to cont-ret pairs. In Experiment 1, cont-enc-ret had a mean score of 0.78 (SD = .08), and cont-ret of 0.66 (SD = .14). A one-way repeated measures ANOVA (RMANOVA), including as covariates the differences between the two conditions in overall recall performance and in temporal score found the main effect of pair category to be significant ($F_{1,26} = 22$; $p < .001$; $\eta^2_p = 0.45$). The equivalent Bayesian ANOVA also found extremely strong evidence in favor of the model including the main effect of pair category with a BF_{10} of > 15,000 in comparison to the null model that included only the covariates as nuisance variables.

In Experiment 2 the relative mean scores were: cont-enc-ret = .75 (SD = .11), cont-ret was = .62 (SD = .19), and cont-enc = .71 (SD = .14). A one-way RMANOVA found the difference between pair categories to be significant ($F_{2,52} = 4.5$; $p < .05$; $\eta^2_p = 0.15$). A Bayesian RMANOVA also found moderate support for the model including pair category over the null model that only included subject number ($BF_{10} = 6.44$). Post-hoc comparisons, with Bonferroni corrections, revealed a significant difference between cont-enc-ret and cont-ret ($t = 2.76$, $p_{bonf} < .05$), all other comparisons were not significant ($p_{bonf} > .35$).

Equivalent absolute order for pairs encoded and retrieved contiguously (cont-enc-ret) over pairs not encoded contiguously (cont-ret)

In order to support our prediction that there is no difference in the absolute score of cont-enc-ret and cont-ret pairs, we used Bayesian statistics that are capable of providing evidence in favor of a null model. To test this hypothesis, we only examined the absolute score of the first item that was ordered, because the second item was typically biased in its ordering by the placement of the first (see Supplemental Material Section D for further discussion). In Experiment 1, the mean absolute score of the first cont-enc-ret pair member was 0.85 (SD = 0.07) and of the first cont-ret pair member was 0.87 (SD = 0.05). A Bayesian paired t-test found anecdotal support for the null hypothesis that there is no difference in absolute score between the pair categories (BF_{01} = 2.3), though it should be noted that cont-ret had a higher score. In Experiment 2, the mean score for cont-enc-ret was .86 (SD = .06), for cont-ret was .85 (SD = .07), and for cont-enc was .86 (SD = .06). A Bayesian RMANOVA found moderate evidence (BF_{01} = 8.8) for the null model that did not include pair category over the model that included pair category. This supports the hypothesis that there is no difference between pair categories in absolute score.

Noting that the absolute scores are close to the maximum value of one and to account for possible ceiling effects, we also examined the absolute deviation that is the unsigned distance between the ordering of an item and its initial position. Importantly, these scores are not close to ceiling (or floor). The results showed a similar pattern to those of the absolute scores. In Experiment 1 cont-enc-ret had a score of 1.6 (SD = .93), and cont-ret had a score of 1.5 (SD = .62). A Bayesian paired t-test found anecdotal evidence for the null hypothesis that there is no difference between the pair categories in absolute deviation (BF_{01} = 2.93). In Experiment 2 the mean scores for cont-enc-ret was 1.6 (SD = .78), cont-ret was 1.5 (SD = .61), and cont-enc was.1.6 (SD = .82). A Bayesian RMANOVA with pair category as the independent variable and absolute deviation as the dependent variable found moderate evidence (BF_{01} = 5.38) in favor of the null model that did not include pair category over the model that did. This further supports the lack of difference in absolute deviation between pair categories.

Equivalent relative score for cont-enc-ret and cont-enc: The contribution of encoding as opposed to recall on relative score

To tease apart the contribution of encoding and recall processes to memory of relative order, in Experiment 2 we introduced an additional pair category, cont-enc. To briefly reiterate,

the cont-enc category includes pairs of items that were encoded contiguously and both items were recalled yet not contiguously. Importantly, they differ from cont-enc-ret only with regard to the recall phase, allowing us to dissociate encoding and recall processes. In Experiment 2 the mean relative score of cont-enc-ret was 0.75 (SD = 0.11), and the mean relative score of cont-enc was 0.71 (SD = 0.14). Post hoc comparisons, with Bonferroni correction, of the ANOVA including pair category found that that there was no significant difference between the two ($t_{(26)} = 1.61$, $p_{bonf} > .35$). The equivalent Bayesian analysis yielded anecdotal evidence in favor of the null model ($BF_{01} = 1.56$) that there is no difference between them.

Improved distance score for cont-enc-ret pairs in comparison to cont-enc pairs

To test our prediction that cont-enc-ret pairs are the basis for formation of temporally structured episodes, we compared their distance score to cont-enc pairs' score. To reiterate, a distance score is the difference between the distance between the items in the ordering phase and their true distance during the study phase. Note, that the comparison between cont-enc-ret and cont-ret pairs is not valid since the latter's range of distance scores include both positive values (i.e. distancing of pair members) and negative values (i.e. bringing them closer). Therefore, we only compared cont-enc-ret to cont-enc—pairs that were studied contiguously but not recalled successively. Both pair categories have a range of only positive values, because it is not possible to order them closer to each other than their actual distance (i.e. zero distance). In line with our prediction, cont-enc-ret pairs were ordered closer to each other than cont-enc pairs, despite their true distance being the same. Thus, cont-enc-ret pairs had a significantly smaller mean distance score than cont-enc pairs (Mean cont-enc-ret = 0.56, SD = 0.09; Mean cont-enc = 0.78, SD = 0.5; $t_{(33)} = -3.02$, $p < .01$, Cohen's $d = -0.53$). The equivalent Bayesian analysis yielded moderate evidence in favor of H1 ($BF_{10} = 8.1$), namely that cont-enc-ret pairs are ordered closer to each other, reflecting their distance into a temporally-structured episode.

References

1. Davelaar, E. J., Goshen-Gottstein, Y., Ashkenazi, A., Haarmann, H. J. & Usher, M. The demise of short-term memory revisited: empirical and computational investigations of recency effects. *Psychol. Rev.* **112**, 3 (2005).

2. Karpicke, J. D. & Roediger, H. L. The Critical Importance of Retrieval for Learning. *Science (80-.).* **319**, 966–968 (2008).

3. Kahana, M. J. Associative retrieval processes in free recall. *Mem. Cognit.* **24**, 103–109 (1996).

4. Rouder, J. N., Morey, R. D., Speckman, P. L. & Province, J. M. Default Bayes factors for ANOVA designs. *J. Math. Psychol.* **56**, 356–374 (2012).

5. Mulligan, N. W. & Lozito, J. P. Order information and free recall: Evaluating the item-order hypothesis. *Q. J. Exp. Psychol.* **60**, 732–751 (2007).

6. Polyn, S. M., Norman, K. A. & Kahana, M. J. A context maintenance and retrieval model of organizational processes in free recall. *Psychological Review* **116**, 129–156 (2009).

7. Miller, G. A. & Chapman, J. P. Misunderstanding analysis of covariance. *J. Abnorm. Psychol.* **110**, 40 (2001).

8. Nairne, J. S., Riegler, G. L. & Serra, M. Dissociative Effects of Generation on Item and Order Retention. *J. Exp. Psychol. Learn. Mem. Cogn.* **17**, 702–709 (1991).
